# EFFECT OF ASPIRIN IN COVID-19 OUTCOMES OF OLDER ADULTS WITH A HISTORY OF CORONARY ARTERY DISEASE

**DOI:** 10.1093/geroni/igac059.3047

**Published:** 2022-12-20

**Authors:** Ali Vaeli Zadeh, Ricardo Criado Carrero, Alan Wong, Noah Mandile, Marina Stukova, German Lopez, Elias Collado, Joshua Larned

**Affiliations:** Holy Cross Health-Jim Moran Cardiovascular Research Institute, Fort Lauderdale, Florida, United States; University of Miami/Holy Cross Hospital, Fort Lauderdale, Florida, United States; Holy Cross Health-University of Miami, Fort Lauderdale, Florida, United States; Jim Moran Cardiovascular Institute, Fort Lauderdale, Florida, United States; University of Miami at Holy Cross, Fort Lauderdale, Florida, United States; University of Miami at Holy Cross, Fort Lauderdale, Florida, United States; Holy Cross Health-Jim Moran Cardiovascular Research Institute, Fort Lauderdale, Florida, United States; Holy Cross Health- University of Miami, Miami, Florida, United States

## Abstract

**Background:**

Advanced age and coronary artery disease (CAD) have been associated with a dismal prognosis in patients infected with COVID-19, most likely due to the virus’s thrombogenic effects. Older adults with a history of CAD have routinely used low-dose Aspirin (LDA) as prevention due to their increased risk of cerebro-cardiovascular events. However, it is unclear if this population would benefit from LDA when infected with COVID-19.

**Methods:**

A retrospective study was conducted using the PearlDiver database (PearlDiver Technologies, Fort Wayne, IN). Using ICD codes, patients aged 65–75 and Elixhauser Comorbidity Score(ECI)>4 with a history of CAD admitted for COVID-19 were identified. The use of LDA for 1 month before the index event was used to split the cohort into two matched groups by age, gender, ECI, and other cardiovascular diseases. Records of groups were reviewed for multiple outcomes 30 days after admission. Pearson’s chi-squared test was used to compare groups. The strength of association was reported as Risk Ratios (RR).

**Results:**

4,017 patients with no difference in the mean age, gender, and ECI were included in each group. No differences present in 30-days all-cause readmission(RR=1.04, CI95% =0.92–1.17, p=0.49), mortality(RR=0.63, CI95%=0.30–1.29, p=0.28), ICU admission(RR=1.01, CI95%=0.89–1.15, p=0.79), gastrointestinal bleeding(RR=1.09, CI95% = 0.85–1.40, p=0.51), and intracranial hemorrhage(RR=0.69, CI95%=0.26–1.83, p=0.62) between groups.

**Conclusion:**

LDA didn’t improve the evaluated outcomes in older persons 30 days after admission. A plausible explanation is that COVID-19’s thrombogenic mechanism is likely atypical.

